# Supplementary material for: A First-in-Human Trial to Evaluate the Safety and Immunogenicity of a G Protein-Based Recombinant Respiratory Syncytial Virus Vaccine in Healthy Adults 18–45 Years of Age
Source: Vaccines (Basel). 2023 May 18;11(5):999. doi: 10.3390/vaccines11050999 (PMC10221330; doi:10.3390/vaccines11050999)
Supplement: Supplementary file 1 [file vaccines-11-00999-s001.zip › vaccines-2250917-supplementary.pdf]

Supplementary File S1. Study vaccine lots

The following lot numbers of the study vaccines and diluents were used: Lyophilized rRSV-G protein 12μg (R201803); CsA diluent 20 μg/mL (C201802); Placebo for rRSV-G-protein (R201701K); Placebo for CsA (C201702K). BARS13 dose level was amended from 10 μg rRSV-G protein and 10 μg Cyclosporine A (CsA) to 9.2 μg rRSV-G protein and 10 μg CsA. During the dosing of 2 sentinel participants in Cohort 1, it was found that RSV-G was constituted in a lower volume than planned (0.42 rather than 0.5 mL) due to needle dead space was not accounted. By agreement between the Sponsor and the phase I site, it was decided to dose with 9.2 μg rRSV-G protein rather than 10 μg, while the total volume remained the same by adding extra diluent.

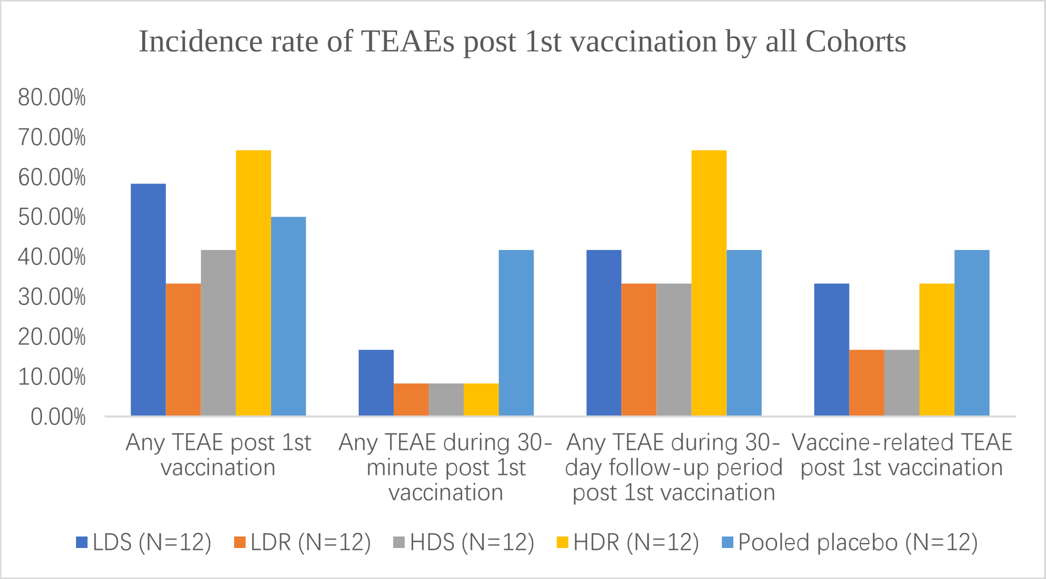

Figure S1. The Incidence Rate of TEAEs post first Vaccination by All Cohorts. Abbreviations: Low dose single (LDS), low dose repeat (LDR), high dose single (HDS), and high dose repeat (HDR).

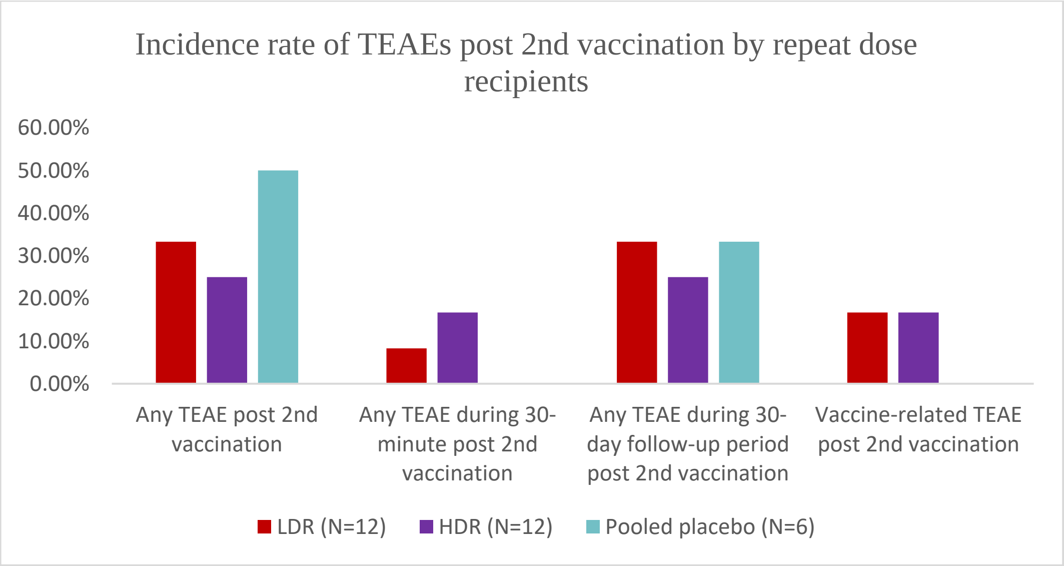

Figure S2. The Incidence Rate of TEAEs post second Vaccination by Repeat Dose Recipients. Abbreviations: Low dose repeat (LDR) and high dose repeat (HDR).
